# Human kidney organoids reveal the role of glutathione in Fabry disease

**DOI:** 10.1038/s12276-021-00683-y

**Published:** 2021-10-15

**Authors:** Jin Won Kim, Hyung Wook Kim, Sun Ah Nam, Jong Young Lee, Hae Jin Cho, Tae-Min Kim, Yong Kyun Kim

**Affiliations:** 1 Cell Death Disease Research Center, College of Medicine, The Catholic University of Korea, Seoul, Korea; 2 Department of Internal Medicine, College of Medicine, The Catholic University of Korea, Seoul, Korea; 3 Department of Internal Medicine, College of Medicine, The Catholic University of Korea, St. Vincent’s Hospital, Suwon, Republic of Korea; 4 Cancer Research Institute, College of Medicine, The Catholic University of Korea and Department of Medical Informatics, College of Medicine, The Catholic University of Korea, Seoul, Korea; 5 Department of Biomedicine & Health Sciences, College of Medicine, The Catholic University of Korea, Seoul, Korea

**Keywords:** Induced pluripotent stem cells, Stem-cell differentiation

## Abstract

Fabry disease is an X-linked lysosomal storage disease caused by a mutation in the galactosidase alpha (GLA) gene. Despite advances in therapeutic technologies, the lack of humanized experimental models of Fabry disease has limited the development of new therapies to cure the disease. Herein, we modeled Fabry disease using human inducible pluripotent stem cell (iPSC)-derived kidney organoids and the CRISPR–Cas9 genome-editing system. GLA-mutant human kidney organoids revealed deformed podocytes and tubular cells with accumulation of globotriaosylceramide (Gb3). Ultrastructural analysis showed abundant electron-dense granular deposits and electron-dense lamellate lipid-like deposits that formed concentric bodies (zebra bodies) in the cytoplasm of podocytes and tubules. The oxidative stress level was increased in GLA-mutant kidney organoids, and the increase was accompanied by apoptosis. Enzyme replacement treatment (ERT) with recombinant human α-Gal A decreased the Gb3 accumulation and oxidative stress, which resulted in amelioration of the deformed cellular structure of the GLA-mutant kidney organoids. Transcription profile analyses showed decreased glutathione (GSH) metabolism in GLA-mutant kidney organoids. GSH replacement treatment decreased oxidative stress and attenuated the structural deformity of the GLA-mutant kidney organoids. GSH treatment also increased the expression of podocyte and tubular markers and decreased apoptosis. In conclusion, GLA-mutant kidney organoids derived from human iPSCs are valuable tools for studying the mechanisms and developing novel therapeutic alternatives for Fabry disease.

## Fabry disease: organoid model suggests possible antioxidant treatment

Kidney organoids harboring the gene mutation that causes Fabry disease provide valuable models for the condition and could inform treatment pathways. Fabry disease is a rare inherited disorder caused by a mutation in the gene for the enzyme galactosidase alpha (GLA). This triggers a build-up of a specific type of fat within cells, which impairs cellular functioning and can cause life-threatening complications. Young Kyun Kim and co-workers at the Catholic University of Korea, Seoul, used human inducible pluripotent stem cells to create kidney organoids with the GLA mutation. Analysis of the organoids revealed deformed epithelial tubular cells and podocytes with dense fat-like deposits inside their cytoplasm. The organoids exhibited high oxidative stress levels, and decreased metabolism of the natural antioxidant, glutathione. The team found that boosting glutathione improved the organoids’ cellular structures and decreased oxidative stress.

## Introduction

Fabry disease is a rare X-linked inherited disorder that causes defects in the glycosphingolipid metabolic pathway that result from the deficient or absent activity of the lysosomal enzyme α-galactosidase A (α-Gal A)^1^. α-Gal A deficiency leads to the accumulation of globotriaosylceramide (Gb3) and related neutral glycosphingolipids within lysosomes, which results in impairment of cellular morphology and function^1,2^. Fabry disease is a multisystemic disease with life-threatening complications such as stroke, heart failure, cardiac arrhythmia, and end-stage renal disease (ESRD) that results in a reduction in life expectancy^3–5^.

Fabry nephropathy results from the accumulation of Gb3 in renal cells, including podocytes, glomerular endothelial cells, mesangial cells, tubular epithelial cells, and vascular endothelial cells^6,7^. Renal involvement is frequent in classic male Fabry disease, as well as a renal variant of nonclassical female Fabry disease^8^. Fabry nephropathy often begins with microalbuminuria or proteinuria in the 2nd to 3rd decade of life^8^. Gradual deterioration of renal function leads to ESRD in the 4th to 5th decade, which is the primary cause of death in patients with untreated Fabry disease^9,10^.

Recombinant enzyme replacement therapy (ERT) using agalsidase-α and agalsidase-β clears cellular deposits of Gb3 and improves disease burden, respectively^11,12^. ERT has become the major therapeutic approach for patients with Fabry disease. ERT improves Fabry-related symptoms and slows or prevents irreversible cardiac or renal damage when started at a relatively early stage^11,12^.

However, ERT has lower therapeutic efficacy when started in advanced stages of Fabry disease^11,12^. ERT is also potentially limited by reaccumulation of Gb3 in podocytes after dose adjustment during the follow-up period and formation of neutralizing antidrug antibodies after infusion, which reduce the efficacy of ERT by increasing cellular Gb3 deposition and results in harmful clinical outcomes that include progressive loss of renal function^13,14^. Thus, adjunct therapy to prevent the progression of renal disease toward ESRD is needed.

Kidney organoids derived from human pluripotent stem cells (hPSCs) contain segmented structures with podocytes, proximal tubules, and distal tubules in nephron-like arrangements and can recapitulate kidney development^15,16^. Advanced technologies for differentiating kidney organoids from hPSCs and efficient genome-editing systems with the clustered regularly interspaced short palindromic repeat CRISPR-Cas9 have enabled modeling of human kidney diseases^15–17^. hPSC-derived kidney organoids that model Fabry disease with CRISPR–Cas9 genome editing of GLA might be useful tools for developing new therapies.

In the present study, we generated GLA-knockout (KO) human inducible pluripotent stem cells (iPSCs) using CRISPR/Cas9-mediated gene editing and differentiated kidney organoids (GLA-KO human iPSC kidney organoids). This work demonstrates that GLA-KO human iPSC kidney organoids phenocopy human Fabry nephropathy and reveals the role of glutathione (GSH) metabolism as a mechanism, as well as an adjuvant therapeutic option for Fabry nephropathy.

## Materials and methods

### CRISPR/Cas9 all-in-one plasmid construction and generation of GLA-KO human iPSCs

All-in-one CRISPR/Cas9 carrying GFP and gRNA was purchased from Life Technologies in California, USA (Cat. A21174, GeneArt CRISPR Nuclease Vector Kit). A human GLA-specific gRNA sequence was provided by Invitrogen Life Technologies (GLA gRNA sequence: TTGGCAAGGACGCCTACCAT). The oligo annealing and subcloning into the Cas9 nuclease reporter vector were performed according to the manufacturer’s instructions. An all-in-one Cas9 nuclease reporter vector expressing Cas9 and, including gRNA against GLA and GFP was transfected by electroporation into iPSCs (CMC11), and the cells were then incubated for 7–10 days. GFP-expressing cells were separated using FACS, seeded on 96-well plates as single cells, and incubated until they pure clones were obtained. A total of 6 clones expressing GFP were obtained and analyzed using Sanger sequencing. Two of the 6 clones, Clones #5 and #9, were identified as having mutations at the genetic lesion targeted using GLA-specific sgRNA-mediated CRISPR/Cas9 and were used for western blot analysis. The chromatograms were analyzed manually, and the mutations were confirmed using immunoblot analysis.

### Kidney organoid differentiation

**Fig. 1:**
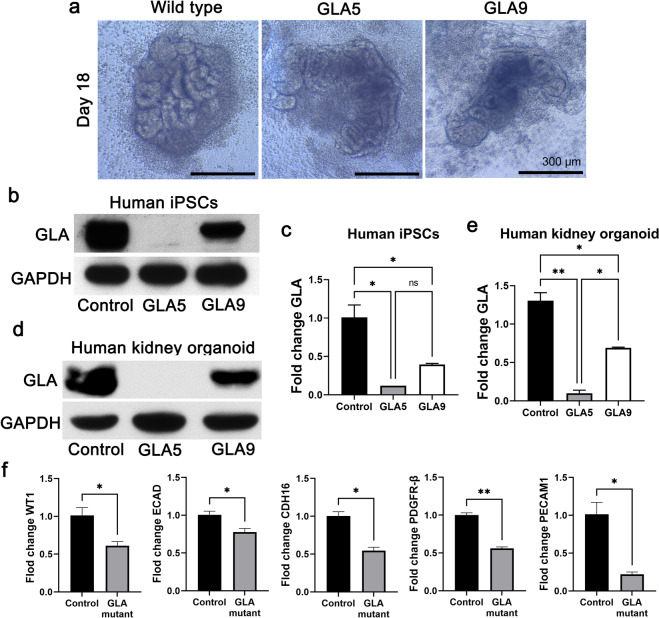
Generation of GLA-mutant iPSC-derived kidney organoids with the CRISPR/Cas9 gene-editing system. **a** Representative brightfield images of the morphology of wild-type kidney organoids and GLA-knockout kidney organoids. Scale bar = 300 μm. **b** Representative western blot of the expression of GLA (Clones #5 and #9) in human iPSCs. **c** qRT–PCR analysis of GLA in human iPSCs. **d** Representative western blot of the expression of GLA (Clones #5 and #9) in human kidney organoids. **e** qRT–PCR analysis of GLA in human kidney organoids. **f** qRT–PCR analysis of WT1, ECAD, CDH16, PDGFR-β, and Pecam1 in human kidney organoids and GLA-mutant organoids. The values are the mean ± SEM. *p < 0.05; **p < 0.01.

The CMC11 iPSC cell line was obtained from The Catholic University of Korea (male donor). Cells were used between passages 30 and 60. Kidney organoid differentiation was performed as previously described^15^. In brief, hPSCs were plated at a density of 5000 cells/well in a 24-well plate in mTeSR1 medium (Stem Cell Technologies, Vancouver, BC, Canada) + 10 µM Y27632 (LC Laboratories, Woodburn, MA) on glass plates (LabTek) coated with 3% GelTrex (Thermo Fisher Scientific, USA) (Day-3). The medium was exchanged with 1.5% GelTrex in mTeSR1 (Day-2), mTeSR1 (Day-1), RPMI (Thermo Fisher Scientific) + 12 µM CHIR99021 (Tocris Bioscience, Bristol, UK) (Day 0), or RPMI + B27 supplement (Thermo Fisher Scientific) (Day-1.5), and the cells were fed every 2–3 days to promote kidney organoid differentiation. The organoids were fixed on Day 18 (Fig. 1a).

### Immunofluorescence and immunohistochemical (IHC) analysis

For immunofluorescence, organoids were fixed on Day 18 unless otherwise noted. For fixation, an equal volume of PBS (Thermo Fisher Scientific) + 8% paraformaldehyde (Electron Microscopy Sciences) was added to the medium for 15 min, after which the samples were washed three times with PBS. The fixed organoid cultures were blocked in 5% donkey serum (Millipore, Billerica, MA, USA) + 0.3% Triton-X-100/PBS, incubated overnight in 3% bovine serum albumin (Sigma-Aldrich, St Louis, MO) + PBS with primary antibodies, washed, incubated with Alexa Fluor-conjugated secondary antibodies (Invitrogen, Carlsbad, CA), washed, and stained with DAPI or mounted in VECTASHIELD H-1000. Images were acquired using a Zeiss LSM 700 confocal microscope (Carl Zeiss, Germany) and ZEN 3.1 software.

For postembedding IHC staining, kidney organoids were embedded in wax after fixation and transversely cut at a thickness of 4 μm using a microtome. Several kidney organoid sections were processed and stained with periodic acid–Schiff (PAS) or TUNEL staining.

The following primary antibodies were used: anti-Gb3 (TCI chemicals, Japan, A2506, 1:1000 dilution), anti-LTL (Vector Labs, Burlingame, CA, FL‐1321, 1:1000 dilution), and anti-NPHS1 (R&D Systems, MN, USA, AF4269, 1:1000). TUNEL staining was performed using an ApopTag Peroxidase In Situ Apoptosis Detection Kit (Millipore), and PAS staining was performed using a Periodic Acid–Schiff Stain Kit (Abcam, ab150680) in accordance with the manufacturer’s instructions.

### Oil red O staining

Organoids were fixed in 4% paraformaldehyde (Electron Microscopy Services), cryoprotected in 30% sucrose solution overnight, and embedded in optimum cutting temperature (OCT) compound (Tissue Tek). The organoids were cryosectioned at 6 µm thickness and mounted on Superfrost slides (Thermo Fisher Scientific). The sections were stained with an oil red O staining kit (Abcam, ab150678) in accordance with the manufacturer’s instructions.

### Mitochondrial staining and reactive oxygen species (ROS) detection

For mitochondrial staining, after washing once with PBS, 5 mM MitoTracker (Thermo Fisher Scientific) and 200 mM Hoechst (Thermo Fisher Scientific) were added to the cells. After incubation for 1 h, a *z*-stack fluorescence image was obtained using a fluorescence microscope.

MitoSOX Red (Thermo Fisher Scientific) was applied to measure the superoxide anion levels. The kidney organoids were treated with Hoechst 33342 (200 mM) for 1 h and MitoSOX Red (5 μM) for 30 min at 37 °C in darkness and washed with PBS, and a z-stack fluorescence image was obtained using a fluorescence microscope.

### Measurement of intracellular calcium potential

For detection of intracellular calcium potential, the kidney organoids were washed with PBS and treated with a Fluo-8 calcium assay kit (5 μM, AAT Bioquest) for 1 h at 37 °C in darkness. Then, the kidney organoids were washed with PBS, and a z-stack fluorescence image was obtained using a fluorescence microscope.

### Electron microscopy (EM) analysis

Kidney organoid samples were fixed in 4% paraformaldehyde and 2.5% glutaraldehyde in 0.1 M phosphate buffer overnight at 4 °C. After washing in 0.1 M phosphate buffer, the samples were postfixed with 1% osmium tetroxide in the same buffer for 1 h at 4 °C. Next, the samples were dehydrated with a series of graded ethyl alcohol solutions, and the ethyl alcohol was exchanged with acetone. The samples were then embedded in Epon 812.

Ultrathin sections (70–80 nm) were obtained using an ultramicrotome (Leica Ultracut UCT, Germany). The ultrathin sections were double-stained with uranyl acetate and lead citrate and then examined under a transmission electron microscope (JEM 1010, Japan) at 60 kV.

For the correlative light and electron microscopy studies, vibratome sections were cryoprotected with 2.3 M sucrose in 0.1 MPB and frozen in liquid nitrogen. Semithin cryosections (2 μm thick) were cut at −100 °C with a glass knife in a Leica EM UC7 ultramicrotome equipped with an FC7 cryochamber (Leica). The sections were labeled at 4 °C overnight using a mouse polyclonal antibody against Gb3 (TCI chemicals A2506, 1:300 dilution). Antibody staining was visualized using Alexa Fluor-conjugated secondary antibodies (Invitrogen). The sections were counterstained with DAPI for 10 min. The coverslipped sections were examined with a confocal microscope and photographed at 200× or 400× magnification with a differential interference contrast setting to find specific areas for later examination by electron microscopy. After the coverslips were floated off the sections, silver enhancement was performed using an HQ silver enhancement kit (Nanoprobes) for 3 min, and the organoids were prepared for electron microscopy as described previously^18,19^.

### Western blot analysis

The kidney organoids were homogenized in boiling lysis buffer (1% SDS, 1 mM sodium orthovanadate, and 10 mM Tris, pH 7.4), and the protein concentration was determined with a BCA Protein Assay Kit (Pierce Biotechnology Inc., Rockford, IL, USA). Equal amounts of the protein were separated on an SDS–polyacrylamide gel. The proteins in the gel were transferred onto an NC membrane. For immunodetection, the blots were incubated overnight in PBS containing 0.1% Tween-20 and 5% skim milk with the primary antibody. The blots were washed and then incubated with a secondary antibody conjugated to horseradish peroxidase (Jackson ImmunoResearch Laboratories, West Grove, PA, USA), and the blots were visualized using a western blotting luminol reagent kit (Santa Cruz Biotechnology, Santa Cruz, CA.).

### qRT–PCR

Kidney organoid samples were harvested, and total RNA from each sample was isolated using an RNAiso Plus Kit (Takara, Japan) according to the manufacturer’s instructions. Complementary DNA was synthesized using a Maxima First Strand cDNA Synthesis Kit for RT-qPCR (Thermo Fisher Scientific, USA). Gene expression was analyzed with Power SYBR Green PCR Master Mix (Applied Biosystems, USA) using real-time PCR (Applied Biosystems, Foster City, CA). The specific primers used were as follows: human GLA, F-5′GACTGGGGAGTAGATCTGCTAAA and R-5′ AGGAGAGCTTTGGCTTGAGG; WT1, F-5′ GCGGAGCCCAATACAGAATA and R-5′ GATGCCGACCGTACAAGAGT; NPHS1, F-5′ GGCTCCCAGCAGAAACTCTT and R-5′CACAGACCAGCAACTGCCTA; PODXL, F-5′ GATAAGTGCGGCATACGGCT and R-5′ GCTCGTACACATCCTTGGCA; PECAM1, F-5′ TCATTACGGTCACAATGACGA and R-5′ GAGTATCTGCTTTCCACGGC; ECAD, F-5′ CGAGAGCTACACGTTCAGG and R-5′ GGGTGTCGAGGGAAAAATAGG; PDGFR-β, F-5′ TGCAGACATCGAGTCCTCCAAC and R-5′ GCTTAGCACTGGAGACTCGTTG; CDH16, F-5′ CCTCATCCTCATTTTCACC and R-5′ GGGCTTCTACTCTGTCCTG; and GAPDH, F-5′ AGGGCTGCTTTTAACTCTGGT and R-5′ CCCCACTTGATTTTGGAGGGA. All qRT–PCR experiments were performed in triplicate, and the relative mRNA expression levels were determined using the 2-ΔΔCt method.

### RNA extraction and GeneChip® Human Gene 2.0 ST Array

Total RNA was extracted following the manufacturer’s instructions. Array hybridization was performed using an Affymetrix GeneChip Human Gene 2.0 ST Array. cDNA was synthesized using the GeneChip WT (Whole Transcript) Amplification kit as instructed by the manufacturer. After cDNA was hybridized to the arrays for 16 h at 45 °C, the chips were processed in a GeneChip Fluidics Station 450 (Affymetrix). Microarray images were collected with the GCS3000 Scanner (Affymetrix), and data were extracted using Affymetrix® GeneChip Command Console® (AGCC) software.

### Microarray expression profiling

Microarray expression profiles were generated using the robust multiarray average method in Affymetrix Power Tools. The microarray data were deposited in the GEO (Gene Expression Omnibus) database with the accession number GSE164941.

### Bioinformatics analyses

GeneSet enrichment analysis (GSEA) (1) was performed with the “C2_cp_KEGG” gene set using GSEA version 4.0 software. GSEA calculated a pathway enrichment score for the highest-ranking genes. The default settings were used.

### Treatment with recombinant human α-Gal A (rhα-GLA)

A previous in vitro study on Fabry disease using mouse aortic endothelial cells from α-Gal A-deficient mice showed that rhα-GLA treatment for 48 hr reduced Gb3 at a dose from 0.1–60 µg/ml^20^. A previous study also demonstrated that a longer incubation time was associated with a lower required dose of the enzyme. Based on a previous study, we modified the dose of the enzyme rhα-GLA in this Fabry kidney organoid model. The enzyme rhα-GLA (Prospec, ENZ-926) was used to treat the GLA-mutant kidney organoids at doses of 3, 6, and 9 µg starting on Day 15 for 3 days at 37 °C under 5% CO2 in an incubator.

### Glutathione treatment and GSH and GSSG detection assay

Glutathione (GSH) was added to GLA-mutant kidney organoids at concentrations of 1, 3, 4, and 5 mM, and the organoids were incubated for 24 h. GSH and GSSG were measured in the kidney organoids using a GSH/GSSG Ratio Detection Assay Kit (Fluorometric—Green) (Abcam, ab138881) according to the manufacturer’s method.

In short, kidney organoids, GLA-mutant kidney organoids, and 1–5 mM GSH-treated GLA-mutant kidney organoids were washed with cold PBS, lysed in 1X mammalian lysis buffer, and centrifuged at 12,000 rpm for 15 min at 4 °C. The clear supernatant was collected. For alternative deproteinization, cold trichloroacetic acid was added. The samples were kept on ice for 10 min and then centrifuged at 12,000 rpm for 5 min at 4 °C. Thereafter, the collected supernatant was neutralized with sodium bicarbonate to pH 4–6. The supernatant was used for GSH and GSSG detection immediately after centrifugation using the GSH/GSSG Ratio Detection Assay Kit.

### Glutathione peroxidase activity assay

Kidney organoid samples were collected and homogenized in cold lysis buffer (50 mM Tris–HCl, 5 mM EDTA and 1 mM dithiothreitol). The samples were centrifuged at 10,000 × *g* for 15 min at 4 °C. The glutathione peroxidase activity in the samples was measured using an absorbance spectrometer at 340 nm using a Glutathione Peroxidase Assay Kit (Cayman Chemicals, Ann Arbor, MI,703102).

## Results

### Generation of GLA-mutant human iPSCs

The coding sequence for GLA was targeted to generate GLA-mutant human iPSCs. GLA-specific single-guide RNA (sgRNA) was prepared to introduce deletion mutations in exon 1 of the GLA gene, as shown in Supplementary Fig. 1a. Human iPSCs were transfected with an all-in-one vector expressing Cas9, sgRNA, and GFP. Two of six clones were identified as having mutations at the genetic lesion targeted using GLA-specific sgRNA-mediated CRISPR/Cas9. Clones #5 and #9 showed deletion of 16 nucleotides and 9 nucleotides in exon 1, causing frameshift, and in-frame mutations, respectively (Supplementary Fig. 1b). The GLA protein in Clone #5 was expected to produce a shorter-than-normal GLA protein due to the early appearance of stop codons.

As expected, the GLA antibody did not detect the GLA protein in Clone #5 compared with the control. Clone #9 had a lower GLA protein level than the control (Fig. 1b). Therefore, we assumed that the disappearance of GLA protein in Clone #5 was due to the early stop codon and that the minimal mRNA expression in Clone #9 resulted in a low amount of GLA protein (Fig. 1c). Thus, GLA-mutant human iPSC cell lines were successfully generated using the CRISP/Cas9 genome-editing system.

### Generation of iPSC-derived kidney organoids recapitulating human Fabry nephropathy

To generate Fabry kidney organoids, GLA-mutant human iPSCs were differentiated into kidney organoids using an adherent cell culture protocol^15,16^. Immunoblotting confirmed the existence of frameshift mutations at the target site and the absence of the corresponding full-length proteins (Fig. 1d). Additionally, we confirmed differences in GLA mRNA expression in differentiated kidney organoids (Fig. 1e). Whether GLA-mutant human iPSC kidney organoids might produce phenotypes relevant to human Fabry nephropathy was examined. Brightfield microscopy revealed that GLA-mutant kidney organoids had deformed structures, and their height was smaller than that of wild-type (WT) kidney organoids (Fig. 1a). Furthermore, the gene expression of podocytes, proximal and distal tubules, pericytes, and endothelial cells was downregulated in GLA-mutant kidney organoids compared to wild-type kidney organoids (Fig. 1f).

**Fig. 2:**
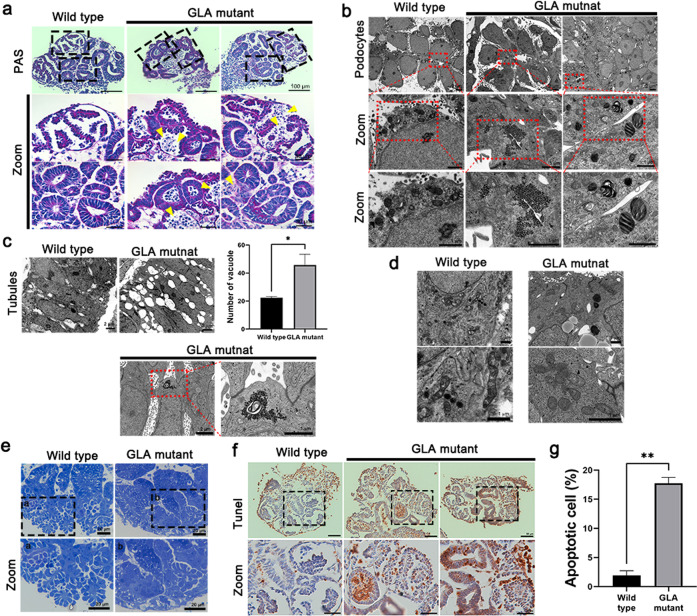
GLA-mutant kidney organoids recapitulated human renal Fabry disease. **a** Representative image of periodic acid–Schiff staining showing glomerular and tubular structures. Scale bar = 100 µm, 30 µm for zoom. **b–d** Representative TEM images showing the zebra bodies in the cytoplasm of podocytes, the vacuoles throughout the cytoplasm of tubules and accumulation of damaged mitochondria and lipid droplets. Scale bar = 2 µm for (**b**, **c**, **d**); 1 µm for (**b**, **c**) zoom. **e** Representative toluidine blue staining images showing podocyte-like structures in wild-type and GLA-mutant kidney organoids. Scale bar = 20 µm. **f** Representative image of TUNEL staining showing apoptotic cells in glomerular and tubular structures. Scale bar = 50 µm, 30 µm for zoom. **g** Quantification of the percentage of apoptotic cells. The values are the mean ± SEM. *p < 0.05; **p < 0.01.

Next, we identified glomerular and tubular-like structures in GLA-mutant kidney organoids. PAS staining showed abundant apoptotic cells in glomerular and tubular structures in GLA-mutant human iPSC kidney organoids (Fig. 2a). Transmission electron microscopy (TEM) showed abundant electron-dense granular deposits and electron-dense lamellate lipid-like deposits that formed concentric bodies (zebra bodies) in the cytoplasm of podocytes and tubules in the GLA-mutant kidney organoids, which were consistent with Fabry disease (Fig. 2b, c). TEM also revealed vacuolation of the tubules (Fig. 2c) and accumulation of damaged mitochondria and lipid droplets in the GLA-mutant kidney organoids (Fig. 2d). Toluidine blue staining was performed, and sections were cut under light microscopy (Fig. 2e). In the WT kidney organoids, the podocytes were arranged intermittently along common basement membrane-like tracks with appropriate spaces between podocytes (Fig. 2e Panel a). In contrast to these structures, deformed podocytes that had fused with neighboring podocytes were observed in the GLA-mutant kidney organoids (Fig. 2e Panel b). TUNEL staining showed abundant apoptotic cells in glomerular and tubular structures in GLA-mutant human iPSC kidney organoids (Fig. 2f, g). These data indicated that the GLA-mutant kidney organoids recapitulated human renal Fabry disease.

### Increased Gb3 expression and lipid accumulation in GLA-KO human iPSC kidney organoids

**Fig. 3:**
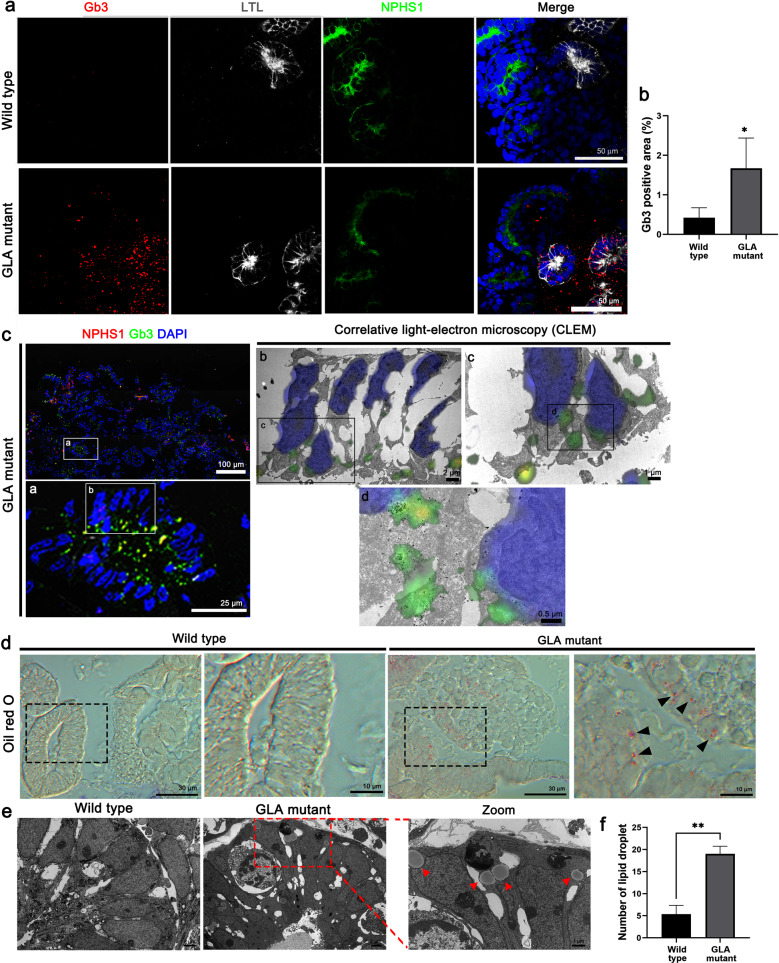
Increased Gb3 expression and lipid accumulation in GLA-KO human iPSC kidney organoids. **a** Representative image of immunofluorescence staining of Gb3, LTL and NPHS1 in WT and GLA-mutant kidney organoids. Scale bar = 50 µm. **b** Quantification of the percentage of Gb3-positive area. **c** Representative images of immunofluorescence staining of NPHS1 and Gb3 overlaid with the corresponding immunogold TEM images labeled with Gb3 obtained from the same field in GLA-mutant kidney organoids. Scale bar = 100 µm for (**c**), 25 µm for (**c**) Panel (**a**), 2 µm for (**c**) Panel (**b**), 1 µm for (**c**) Panel (**c**), and 0.5 µm for (**c**) Panel (**d**). **d** Representative oil red O staining images showing lipid droplet formation in podocytes and tubules. Scale bar = 30 µm, 10 µm for zoom. **e** Representative TEM images showing lipid droplets (red arrowhead). Scale bar = 2 µm 1 µm for e panel. **f** Quantification of the number of lipid droplets. The values are the mean ± SEM. *p < 0.05; **p < 0.01.

Next, to determine the accumulation of Gb3, a main pathological mechanism in human Fabry disease^6,7^, confocal microscopy was performed, which revealed extensive accumulation of Gb3 in podocytes and tubular cells in GLA-mutant human iPSC kidney organoids (Fig. 3a, b). To precisely determine the ultrastructural localization of Gb3 in the Fabry kidney organoids, correlative light microscopy and immunogold EM with Gb3 labeling were performed. Overlay of confocal microscopy and immunogold EM images confirmed that intracellular Gb3 puncta in tubule-like cells corresponded to silver-enhanced immunogold particle labeling for Gb3 in GLA-mutant human iPSC kidney organoids (Fig. 3c).

Renal lipid accumulation is often observed in lysosomal storage diseases such as human Fabry disease^21^. Increased lipid droplet formation in podocytes and tubules was also observed in GLA-KO human iPSC kidney organoids based on oil red O staining and ultrastructural analyses (Fig. 3d–f). Taken together, these findings indicate that GLA-mutant kidney organoids have the ability to recapitulate human renal Fabry disease phenotypes.

### ERT attenuated oxidative stress and attenuated the structural and transcriptional changes in GLA-mutant human iPSC kidney organoids

ERT is a major therapeutic option for patients with Fabry disease that improves Fabry-related symptoms and slows renal damage when started at a relatively early stage^11,12^. Thus, we investigated whether the therapeutic efficacy of enzyme replacement with the enzyme rhα-GLA could be recapitulated in the Fabry kidney organoid model. Fabry kidney organoids were treated with the enzyme rhα-GLA at concentrations of 3, 6, and 9 µg/ml for 3 days.

**Fig. 4:**
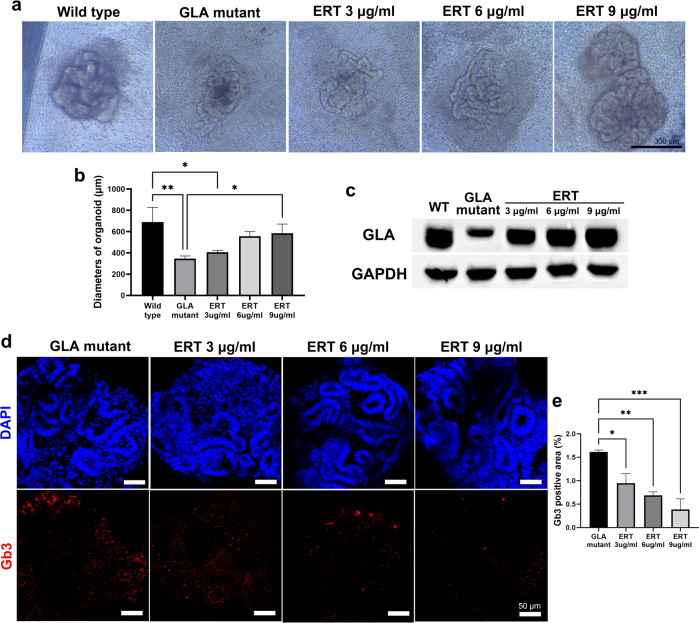
ERT ameliorated the structural changes and reduced the accumulation of Gb3 in GLA-mutant human iPSC kidney organoids. **a** Representative brightfield image of changes in the morphology of GLA-mutant kidney organoids after ERT. Scale bar = 300 µm. **b** Quantification of kidney organoid diameters after ERT. **c** Representative western blot of the expression of GLA after ERT. **d** Representative image of immunofluorescence staining of Gb3 in GLA-mutant kidney organoids after ERT. Scale bar = 50 µm. **e** Quantification of the percentage of Gb3-positive area. The values are the mean ± SEM. *p < 0.05; **p < 0.01; ***p < 0.001.

ERT restored the deformed cellular structure of Fabry kidney organoids to a more organized pattern and increased cell viability to a level similar to that of WT organoids (Fig. 4a). The decrease in size of the GLA-KO human iPSC kidney organoids was attenuated in accordance with ERT (Fig. 4b). ERT resulted in increased GLA protein expression in GLA-KO human iPSC kidney organoids, restoring it to a level similar to that in WT kidney organoids (Fig. 4c). The accumulation of Gb3 was decreased in a dose-dependent manner after ERT, although accumulation of Gb3 remained after ERT (Fig. 4d, e).

We hypothesized that increased oxidative stress and reactive oxygen species (ROS) levels, similar to Gb3 accumulation, are involved in renal remodeling in Fabry disease^22–28^. This hypothesis was investigated in the Fabry kidney organoid model. Mitochondria are the main source of ROS generation, and mitochondrial DNA is a main target of ROS.

**Fig. 5:**
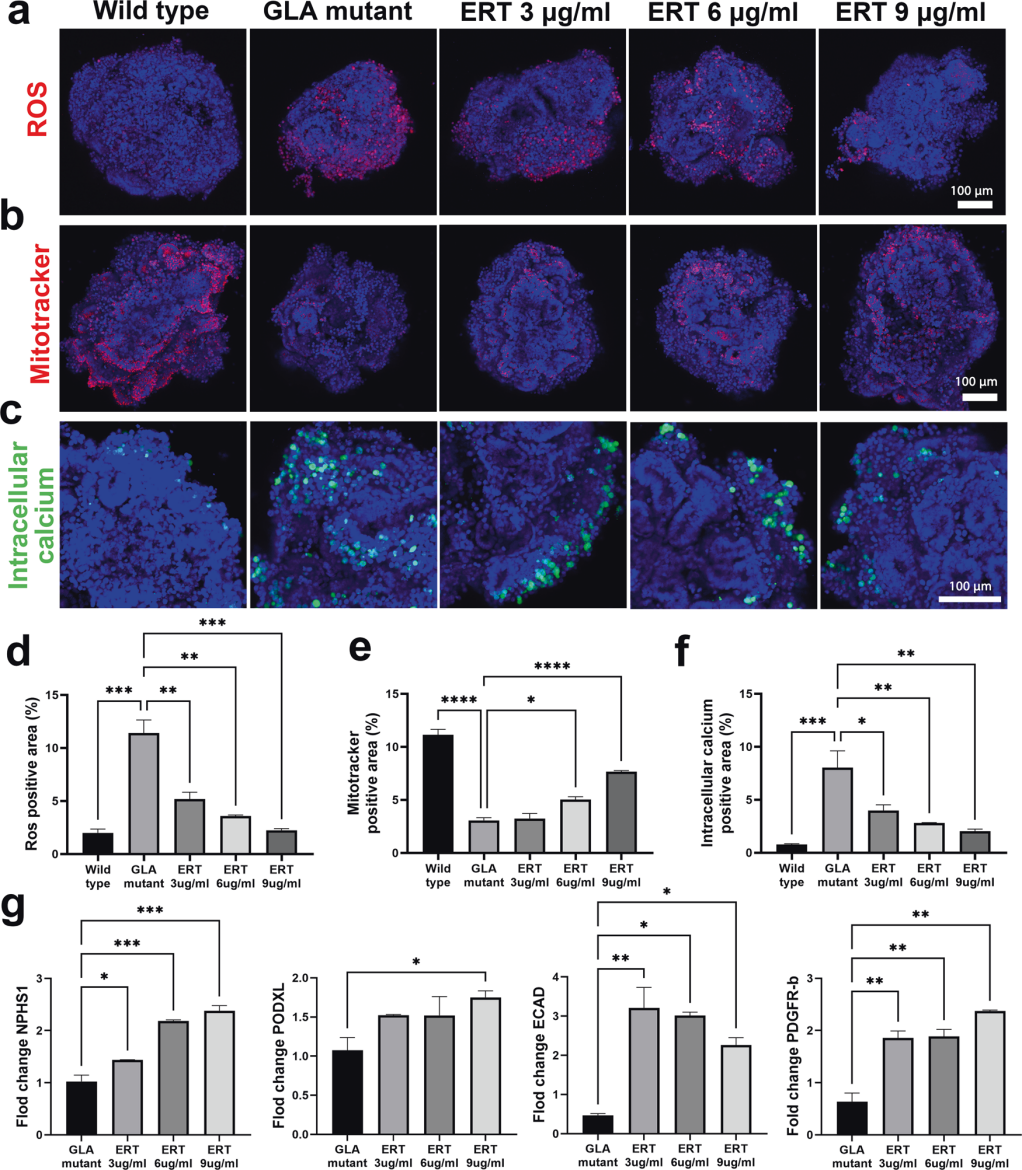
ERT relieves oxidative stress and attenuates the transcriptional changes in GLA-mutant human iPSC kidney organoids. **a** Representative image of immunofluorescence staining with MitoSOX Red. Scale bar = 50 µm. **b** Representative images of immunofluorescence staining with MitoTracker. Scale bar = 50 µm. **c** Representative images of immunofluorescence staining with Fluo-8 showing the levels of intracellular calcium. Scale bar = 50 µm. **d** Quantification of the fluorescence intensity of MitoSOX Red. **e** Quantification of the fluorescence intensity of MitoTracker. **f** Quantification of intracellular calcium via analysis of the fluorescence intensity of Fluo-8. **g** qRT–PCR analysis of NPHS1, PODXL, ECAD, and PDGFR-β. The values are the mean ± SEM. *p < 0.05; **p < 0.01; ***p < 0.001.

The superoxide in mitochondria was examined using MitoSOX Red staining. MitoSOX Red is oxidized by superoxide in mitochondria, resulting in the emission of red fluorescence. MitoSOX fluorescence was increased in Fabry kidney organoids but was decreased after ERT (Fig. 5a, d). The intensity of MitoTracker fluorescence was decreased in Fabry kidney organoids but recovered after ERT, although it remained weaker than that in WT kidney organoids (Fig. 5b, e). These findings indicated that mitochondrial oxidative stress was increased in the Fabry kidney organoids.

Oxidative stress and ROS alter ion transporters, which leads to changes in the second messenger system and primary calcium homeostasis, resulting in an increase in calcium influx into the cytoplasm. In the present study, staining with the calcium-sensitive fluorescent dye Fluo-8 was used to examine intracellular calcium. The numbers of Fluo-8-positive cells were increased in Fabry kidney organoids. After ERT, intracellular calcium was decreased but was still greater than that in WT kidney organoids (Fig. 5c, f).

Gene expression of renal cells in kidney organoids, such as podocytes and tubular epithelial cells, was increased after ERT in GLA-KO human iPSC kidney organoids (Fig. 5g).

Our data showed that increased oxidative stress and mitochondrial dysfunction were involved in the pathogenesis of Fabry disease and were not fully eliminated even with ERT. Because ERT clinically improves the disease and symptoms but is suboptimal due to the occurrence of irreversible renal injury in cases of late diagnosis^22^, our findings are compatible with the natural clinical course of human Fabry disease.

### Transcriptomic analyses revealed the mechanisms of renal Fabry disease

Gene expression profiles were obtained for GLA-mutant Fabry kidney organoids (*n* = 3) and WT kidney organoids (*n* = 2). To elucidate the transcriptional program associated with GLA-mutant kidney organoids and the consequences, 100 differentially expressed genes (DEGs) were first identified on the basis of fold change values (50 genes were upregulated and downregulated in GLA-mutant Fabry kidney organoids compared with WT kidney organoids; Supplementary Table 1). The DEGs downregulated in GLA-mutant Fabry kidney organoids included various genes involved in inflammation and oxidative stress. For example, glutathione S-transferase mu 5 (GSTM5) encodes the mu class of glutathione S-transferases with roles in the detoxification of various types of toxins^29–31^. Cathepsin G (CTSG) plays an important role in the process of inflammation and promotes the migration of neutrophils, monocytes, and antigen-presenting cells^32^.

**Fig. 6:**
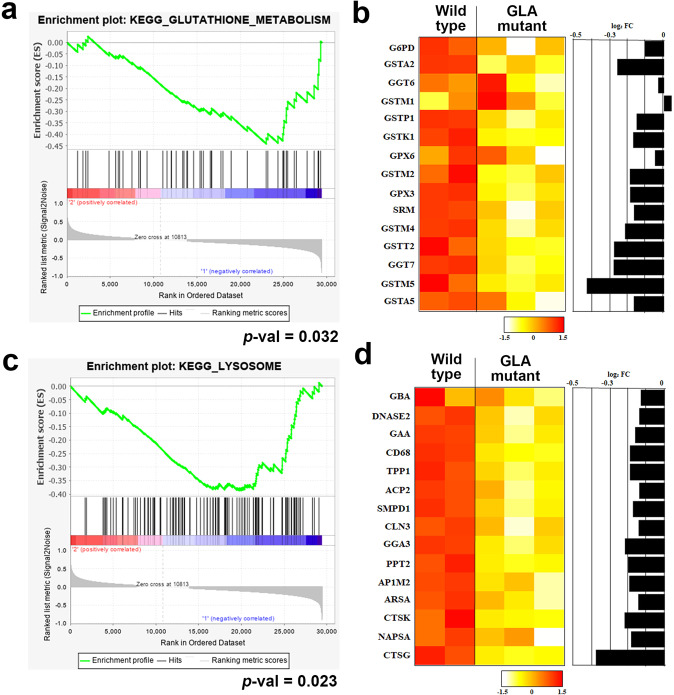
Transcriptional changes in functionally coordinated genes between WT kidney organoids and GLA-mutant kidney organoids. **a** Enrichment plot of the GSEA results in “KEGG_GLUTATHIONE_METABOLISM”. **b** Heatmap of the top 15 leading-edge genes in the gene list of the GSEA results in “KEGG_GLUTATHIONE_METABOLISM”. **c** Enrichment plot of the GSEA results in “KEGG_LYSOSOME”. **d** Heatmap of the top 15 leading-edge genes in the gene list of the GSEA results in “KEGG_LYSOSOME”.

To further investigate the transcriptional changes in functionally coordinated genes, Gene Set Enrichment Analysis (GSEA)^33^ was performed using the Kyoto Encyclopedia of Genes and Genomes (KEGG) functional gene sets available in MSigDB (https://www.gsea-msigdb.org/gsea/msigdb). The top 10 downregulated and 10 upregulated KEGG gene sets in GLA-mutant Fabry kidney organoids compared with WT kidney organoids are shown in Supplementary Table 2. Among the gene sets, genes with roles in GSH metabolism (“KEGG_GLUTATHIONE_METABOLISM”, *p* value, 0.032) were substantially downregulated in Fabry kidney organoids, indicating that the molecular functions were relatively deactivated. An enrichment plot and an expression heatmap of the leading-edge genes are shown in Fig. 6a and b. In addition to GSTM5, various subunits of glutathione S-transferase and gamma-glutamyltransferase were consistently downregulated in Fabry kidney organoids. In addition, lysosomal genes involved in lysosomal synthesis and hydrolytic enzymes were coordinately downregulated in Fabry kidney organoids (*p* value, 0.023). Enrichment plots and leading-edge genes of ‘KEGG_LYSOSOME” are presented in Fig. 6c and d, which show the genes that were coordinately downregulated in Fabry kidney organoids with potential lysosomal dysfunction.

### GSH treatment ameliorated renal Fabry disease

**Fig. 7:**
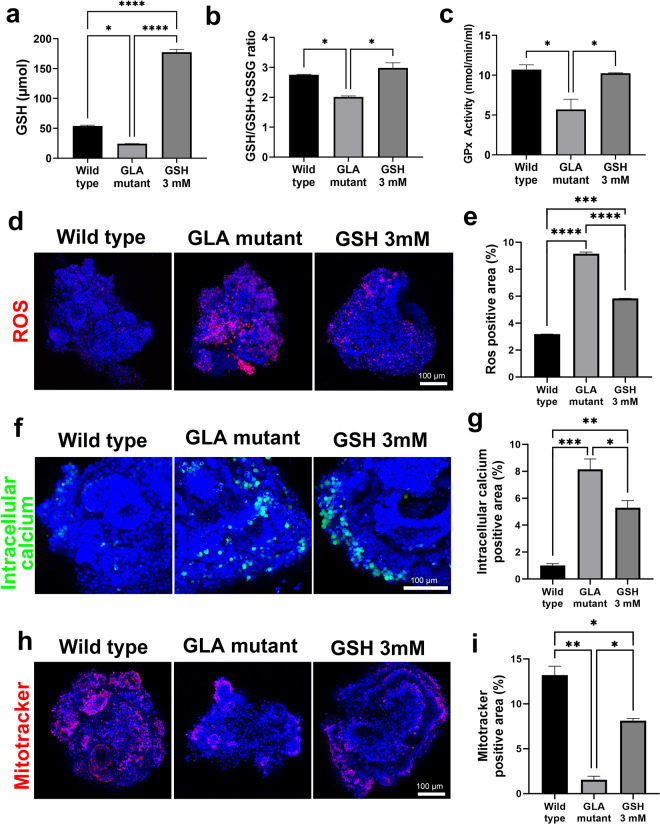
GSH treatment reduces oxidative stress in Fabry kidney organoids. **a** GSH, **b** GSH/GSH + GSSG and **c** GPx were measured in GLA-mutant and GSH-treated GLA-mutant kidney organoids. **d** Immunofluorescence image showing the levels of ROS by staining with MitoSOX Red. Scale bar = 50 μm. **e** Quantification of ROS via analysis of the fluorescence intensity of MitoSOX Red. **f** Immunofluorescence image showing the levels of intracellular calcium by staining with Fluo-8. Scale bar = 50 μm. **g** Quantification of intracellular calcium via analysis of the fluorescence intensity of Fluo-8. **h** Immunofluorescence image showing mitochondrial dysfunction revealed by MitoTracker staining. Scale bar = 50 μm. **i** Quantification of mitochondrial levels via analysis of the fluorescence intensity of MitoTracker. The values are the mean ± SEM. *p < 0.05; **p < 0.01; ***p < 0.001; ****p < 0.0001.

Based on transcriptomic analyses, we hypothesized that GSH treatment reduced oxidative stress and ROS levels, which resulted in the improvement of Fabry disease. To choose the optimal dose of GSH for the in vitro experiment, we evaluated the efficacy of GSH at various doses from 1 to 5 mM (Supplementary Fig. 2). The gene expression of podocyte markers in Fabry kidney organoids was most increased after GSH treatment at a dose of 3 mM (Supplementary Fig. 2). Therefore, we chose 3 mM as the experimental dose of GSH for the Fabry kidney organoid model. The reductions in GSH levels in Fabry kidney organoids were attenuated after GSH treatment (Fig. 7a). The GSH/total glutathione (GSH + GSSG) ratio of Fabry kidney organoids recovered to a level similar to that of WT kidney organoids (Fig. 7b). Glutathione peroxidase (GPx) protects against apoptosis in response to oxidative damage by catalyzing the reduction of both organic and hydrogen peroxides. The reduction in GPx activity in Fabry kidney organoids was attenuated after GSH treatment (Fig. 7c). GSH treatment decreased MitoSOX fluorescence and cytoplasmic intracellular calcium levels (Fig. 7d–g). The intensity of MitoTracker fluorescence in Fabry kidney organoids increased after GSH treatment (Fig. 7h-i). These findings indicated that GSH treatment increased GSH levels in Fabry kidney organoids, which decreased ROS and oxidative stress levels.

**Fig. 8:**
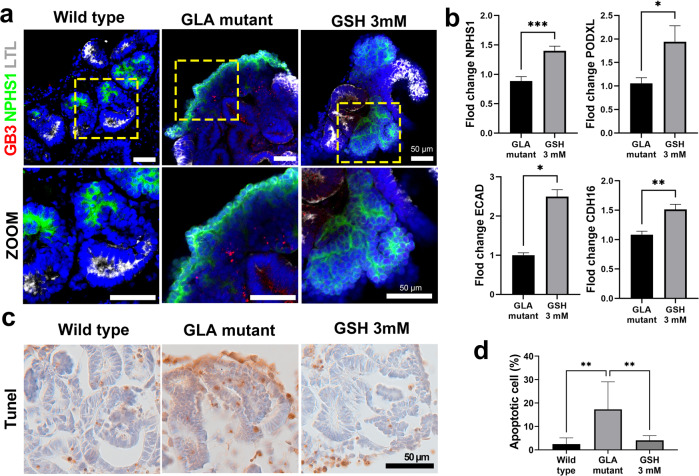
Structural and functional changes caused by GSH treatment in GLA-mutant kidney organoids. **a** Immunofluorescence staining of globotriaosylceramide (Gb3) and markers of podocytes (NPHS1) and proximal tubules (LTL) in GLA-mutant kidney organoids and GSH-treated GLA-mutant kidney organoids. Scale bar = 50 µm. **b** qRT–PCR analysis of NPHS1, PODXL, ECAD, and CDH16. **c** TUNEL staining images showing apoptotic cells. Scale bar = 50 µm. **d** Quantification of the percentage of apoptotic cells. The values are the mean ± SEM. *p < 0.05; **p < 0.01; ***p < 0.001.

Immunofluorescence analysis showed that GSH treatment caused glomerular-like structures to be organized in a pattern similar to that in WT kidney organoids, while the glomerular-like structures were linear and unorganized in Fabry kidney organoids (Fig. 8a). GSH treatment increased the mRNA expression of podocyte and tubular markers (Fig. 8b). TUNEL staining showed that apoptotic cell death in Fabry kidney organoids decreased after GSH treatment (Fig. 8c, d). Taken together, these findings indicate the efficacy of GSH as a rescue therapy for Fabry disease.

## Discussion

To understand the mechanism of Fabry disease, in vivo and in vitro models recapitulating human Fabry disease are essential. The α-Gal A KO mouse model, the first in vivo model of Fabry disease, showed a marked increase in Gb3 in the kidneys^34^. However, the mouse model does not mimic the classic male renal phenotype because only small-fiber neuropathy and hypertrophic cardiomyopathy are the key features of the model^6,34^. Therefore, investigation of the mechanisms and assessment of treatment efficacy using this mouse model of Fabry disease may be limited^6,34^. To overcome this limitation and develop new therapeutic strategies, generating an experimental model reflecting human renal Fabry disease is essential.

In the present study, an in vitro disease model of renal Fabry disease was developed. The present study was performed with kidney organoids derived from human iPSCs, which provide several advantages as disease models. Given the advancements in genome-editing technology obtained with the CRISPR/Cas9 system, human Fabry disease can be readily modeled in a pathologically and genetically relevant manner. The model used in this study phenocopied accumulation of Gb3 in podocytes, renal tubular epithelial cells and zebra bodies as pathognomonic findings of human Fabry disease.

One of the clinical issues associated with renal Fabry disease is the low therapeutic efficacy of ERT when treatment is started in the advanced stages^11,12^. New strategies, including new forms of ERT, pharmacological chaperone therapy, substrate reduction therapy, and gene therapy, have been studied over the past decade.

This study demonstrated that ERT not only ameliorated structural deformities but also reduced oxidative stress and increased the gene expression of kidney cells in Fabry kidney organoids, which means that Fabry kidney organoids are useful tools for investigating the efficacy of ERT and will contribute to the development of new forms of ERT.

Recently, identification of new molecules through high-throughput screening has emerged as an attractive approach to search for new pharmacological chaperone therapies for Fabry disease^35^. Kidney organoids derived from hPSCs have shown great potential in high-throughput screening^36^. Freedman and colleagues established an automated high-throughput screening system for polycystic kidney disease (PKD) based on kidney organoid differentiation. They demonstrated that blebbistatin, a specific inhibitor of nonmuscle myosin II, induced a significant increase in cyst formation of PKD1-mutant kidney organoids, which provides an important clue for the development of new treatments for PKD^36^. In this context, GLA-mutant kidney organoids can also be applied for high-throughput screening systems for Fabry disease. This strategy will help shorten the time required for treatment development and will reduce costs^35^.

In the present study, we used another strategy for treatment development: transcriptomic analysis. We demonstrated that the in vitro kidney organoid model enabled the identification of a novel mechanism that may be used for the treatment of Fabry disease. GSH, an endogenous low-molecular-weight thiol-containing compound, plays an essential role in cellular redox reactions and protects cells from ROS^37^. Under oxidative stress, GSH is oxidized to glutathione disulfide (GSSG), and redox reactions are catalyzed by glutathione peroxidase (GPx) and GSSG reductase^38^. The kidneys are highly dependent on an adequate supply of GSH to maintain normal function. In addition, the kidneys are potentially exposed to high concentrations of oxidants. Maintenance of the mitochondrial GSH pool is critical for cellular and mitochondrial redox homeostasis and important in determining susceptibility to ROS^39^.

Although most cells can catalyze GSH synthesis, rates of GSH production may be inadequate or insufficient to maintain cellular concentrations of GSH during disease or toxic states^39^. In clinical studies, the GSH concentration in whole blood is dramatically decreased in patients with chronic kidney disease, possibly because of severe impairment of GPx and GSSG reductase activity^38^. Renal cellular concentrations of GSH are maintained by both the intracellular synthesis and transport from outside the cells^39^.

In the present study, GSH metabolism was shown to be decreased in a Fabry disease model based on transcriptomic analysis, and restoring GSH reduced oxidative stress from ROS and improved cellular damage in Fabry disease. These data provide novel insights into treatment options for Fabry disease.

In conclusion, the data from the present study reveal the usefulness of GLA-KO human iPSC kidney organoids as an efficient model of human renal Fabry disease. GLA-KO human kidney organoids recapitulate human renal Fabry disease and might be valuable tools for studying the mechanisms and for the development of novel therapeutic alternatives for Fabry disease. The described methodologies are broadly applicable and adaptable to Fabry disease involving diverse tissues, including the heart, brain, and skin, and can be used immediately to experimentally investigate molecular pathways and novel treatments.

## Supplementary information

Supplementary Information
