# Supplementary material for: Human kidney organoids reveal the role of glutathione in Fabry disease
Source: Exp Mol Med. 2021 Oct 15;53(10):1580–91. doi: 10.1038/s12276-021-00683-y (PMC8568890; doi:10.1038/s12276-021-00683-y)
Supplement: Supplementary file 1 — Supplementary Information [file 12276_2021_683_MOESM1_ESM.pdf]

Supplementary Information for:

Human kidney organoids reveal the role of glutathione in Fabry disease

Jin Won Kim[1*], Hyung Wook Kim[1,2,3*], Sun Ah Nam[1], Jong Young Lee [1], Hae Jin Cho,[4,5] Tae-Min Kim,[4,5] Yong Kyun Kim[1,2,3†]

[1]Cell Death Disease Research Center, College of Medicine, The Catholic University of Korea, Seoul, Korea,

[2]Department of Internal Medicine, College of Medicine, The Catholic University of Korea, Seoul, Korea,

[3]Department of Internal Medicine, College of Medicine, The Catholic University of Korea, St. Vincent's Hospital, Suwon, Republic of Korea,

[4]Cancer Research Institute, College of Medicine, The Catholic University of Korea and Department of Medical Informatics, College of Medicine, The Catholic University of Korea,

[5]Department of Biomedicine & Health Sciences, College of Medicine, The Catholic University of Korea

Supplementary Figures

Supplementary Figure 1 GLA knockout human iPSCs generated using CRISPR-Cas9. a Schematic diagram of the single-guide RNA targeting site located exon1 of GLA gene. **b** Clones #5 and #9 Sequence after the knockout GLA target gene

Supplementary Figure 2 Gene expression of podocyte makers of GLA mutant kidney organoids treated with GSH by concentration qRT-PCR analysis of NPHS1, WT1 and PODXL (podocyte markers) at GLA mutant kidney organoids with 1 mM to 5mM GSH.

Supplementary Figure 1

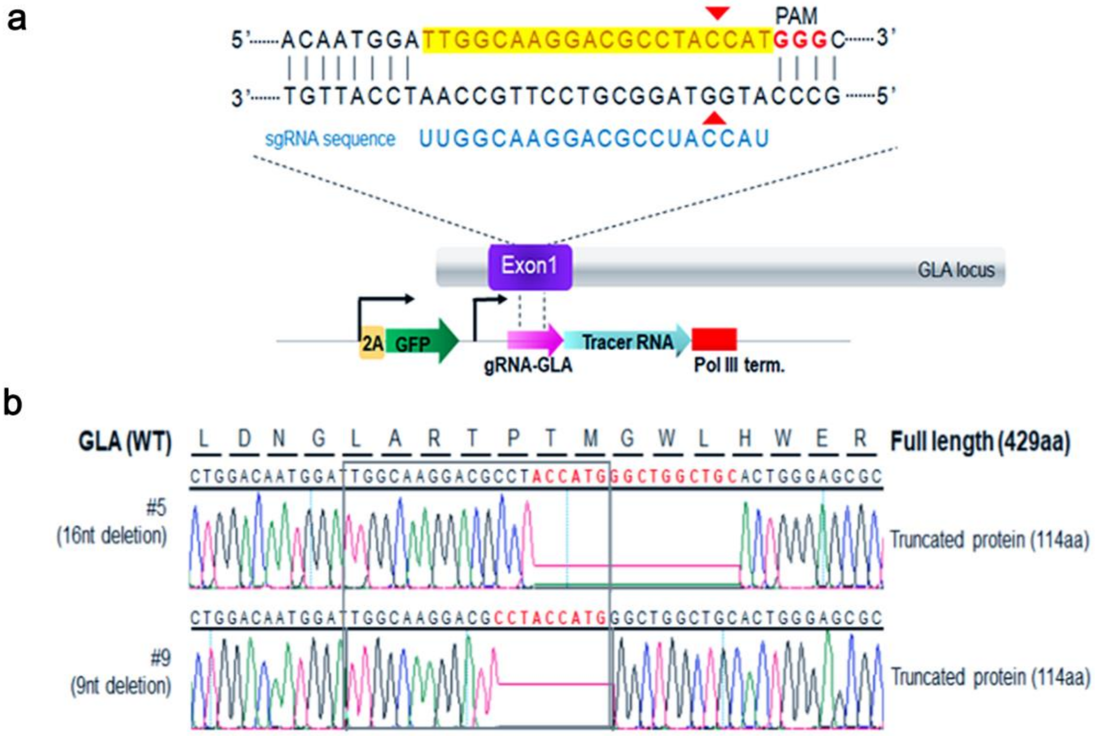

Supplementary Figure 2

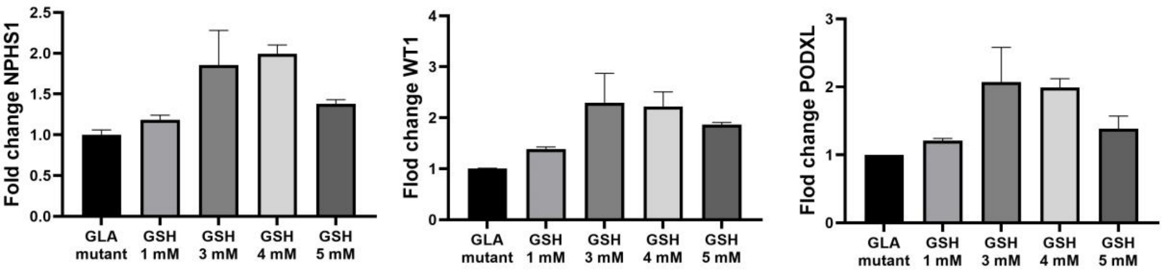

Supplementary Tables

Supplementary Table 1 Top 50 Gene list of log_2 Fold change

TYPE	Gene name	log2 Fold change	*p*-value
	EGR1	-1.004	0.00124
	CHCHD2	-0.878	0.0293
	LOC101927372	-0.736	0.00217
	ZNF585B	-0.582	0.07527
	ZNF229	-0.57	0.00281
	FOS	-0.556	0.01384
	LPAL2	-0.541	0.06027
	BCRP3	-0.539	0.01006
	LBX1	-0.529	0.00227
	MIR519A1	-0.491	0.04004
	LOC644919	-0.49	0.00501
	HIC1	-0.487	0.01558
	MIR4653	-0.468	0.07066
	MIR4779	-0.463	0.00458
	MIR4490	-0.459	0.04868
	MIR221	-0.457	0.00743
	SNORD124	-0.452	0.12936
	MIR3926-2	-0.445	0.00361
	GSTM5	-0.445	0.153
	MIR514B	-0.441	0.12027
	CES4A	-0.438	0.00287
	KIAA0895L	-0.43	0.00138
**Down regulate**	LOC105369895	-0.426	0.01003
	CCL23	-0.423	0.01106
	OR4A5	-0.423	0.02728
	MIR3188	-0.42	0.04245
	KRTAP5-4	-0.417	0.01896
	AOC2	-0.415	0.00954
	MIR4661	-0.414	0.18795
	TGFB1I1	-0.412	0.00043
	UBE2Q2P1	-0.411	0.01276
	LOC100506470	-0.406	0.01841
	LOC440300	-0.406	0.26228
	MIR4497	-0.4	0.21974
	RNF208	-0.399	0.02241
	LYZL2	-0.399	0.19027
	FOSB	-0.39	0.10266
	LOC100500773	-0.388	0.18784
	MSTO2P	-0.387	0.00357
	SNORD116-18	-0.386	0.01204
	CTSG	-0.386	0.04886
	KRTAP5-5	-0.384	0.18508
	HOXD10	-0.384	0.00345
	MIR520B	-0.382	0.2236
	SPRY4	-0.38	0.00996
	LOC105374468	-0.38	0.00284
	LOC440173	-0.378	0.01542
	LOC102723832	-0.374	0.07459
	TNS2	-0.374	0.0202
	FAM86JP	-0.373	0.045

TYPE	Gene name	log2 Fold change	*p*-value
	MIR18B	0.842	0.00053
	RNU5D-1	0.722	0.00258
	LOC105376438	0.707	0.00078
	SNORA79	0.698	0.07678
	LOC105369508	0.687	0.06874
	SCARNA15	0.674	0.0276
	LOC105375032	0.65	0.00657
	SNORA72	0.641	0.00031
	MIR548F2	0.608	0.0009
	SNORD103C	0.592	0.00493
	LOC105369507	0.582	0.04897
	MIR548H1	0.555	0.11592
	SCARNA1	0.552	0.00593
	RNU5E-1	0.541	3.00E-05
	SNORD90	0.532	0.13779
	SCARNA23	0.521	0.06656
	TAS2R50	0.516	0.01066
	MIR363	0.514	0.05867
	PMCH	0.5	0.02111
	SNORD116-25	0.5	0.00042
	SNORD63	0.498	0.01327
	MIR1284	0.492	0.00366
	MIR19B2	0.48	0.14025
**Up regulate**	LOC101927055	0.473	0.02
	MIR4802	0.461	0.02749
	MIR1206	0.459	0.05769
	RNU5B-1	0.458	0.14358
	EVI2A	0.451	0.00763
	TAS2R30	0.45	0.00999
	MIR4782	0.44	0.49703
	MIR603	0.438	0.05457
	SCARNA8	0.436	0.01794
	PRH2	0.429	0.01097
	LOC105373257	0.427	0.00072
	SNORA65	0.427	0.00433
	MIR374A	0.423	0.13185
	UBE2CP5	0.419	0.00145
	LOC101927827	0.418	0.02219
	MIR4499	0.407	0.04382
	DLEU2L	0.402	0.08457
	C9orf84	0.4	0.01799
	TRAJ21	0.399	0.13483
	MIR487A	0.398	0.05652
	MIR181A1	0.398	0.08131
	TAS2R20	0.398	0.02409
	MIR1278	0.394	0.0005
	SNORD16	0.394	0.00037
	HIF1A-AS2	0.39	0.00697
	LOC105370765	0.389	0.025
	LRRC19	0.388	0.02835

Supplementary Table 2 Top 10 downregulated and 10 upregulated leading-edge genes in GLA-mutant Fabry kidney organoids compared with WT kidney organoids

TYPE	NAME	ES	NES	p-val	FDR
**Downregulated**	KEGG_GLUTATHIONE_METABOLISM	-0.44	-1.44	0.032	0.309
	KEGG_JAK_STAT_SIGNALING_PATHWAY	-0.36	-1.36	0.023	0.31
	KEGG_LYSOSOME	-0.39	-1.43	0.005	0.313
	KEGG_ADHERENS_JUNCTION	-0.4	-1.37	0.04	0.316
	KEGG_ETHER_LIPID_METABOLISM	-0.46	-1.37	0.072	0.316
	KEGG_CALCIUM_SIGNALING_PATHWAY	-0.36	-1.37	0.011	0.318
	KEGG_THYROID_CANCER	-0.5	-1.46	0.049	0.319
	KEGG_GNRH_SIGNALING_PATHWAY	-0.4	-1.42	0.02	0.322
	KEGG_SYSTEMIC_LUPUS_ERYTHEMATOSUS	-0.42	-1.39	0.041	0.322
	KEGG_TOLL_LIKE_RECEPTOR_SIGNALING_PATHWAY	-0.38	-1.38	0.034	0.322
**Upregulated**	KEGG_PROTEIN_EXPORT	0.49	1.69	0.013	0.153
	KEGG_TASTE_TRANSDUCTION	0.35	1.48	0.016	0.32
	KEGG_ASCORBATE_AND_ALDARATE_METABOLISM	0.46	1.47	0.061	0.225
	KEGG_PENTOSE_AND_GLUCURONATE_INTERCONVERSIONS	0.38	1.28	0.157	0.568
	KEGG_FATTY_ACID_METABOLISM	0.28	1.1	0.273	1
	KEGG_NICOTINATE_AND_NICOTINAMIDE_METABOLISM	0.31	1.1	0.336	1
	KEGG_STARCH_AND_SUCROSE_METABOLISM	0.27	1.08	0.263	0.962
	KEGG_NITROGEN_METABOLISM	0.28	0.94	0.558	1
	KEGG_RETINOL_METABOLISM	0.22	0.94	0.638	1
	KEGG_DRUG_METABOLISM_OTHER_ENZYMES	0.22	0.91	0.636	1
